# Discovery and identification of the prognostic significance and potential mechanism of FMO2 in breast cancer

**DOI:** 10.18632/aging.205204

**Published:** 2023-11-13

**Authors:** Lichun Wu, Jie Chu, Lijuan Shangguan, Mingfei Cao, Feng Lu

**Affiliations:** 1 Department of Clinical Laboratory, Sichuan Cancer Hospital and Institute, Sichuan Cancer Center, School of Medicine, University of Electronic Science and Technology of China, Chengdu, China; 2 The First People’s Hospital of Ziyang, Ziyang, China; 3 Outpatient Department, People’s Hospital of Jianyang, Jianyang, China; 4 Department of Clinical Laboratory, Chuankong Hospital of Jianyang, Jianyang, China; 5 Department of Experimental Medicine, The People’s Hospital of Jianyang City, Jianyang, China

**Keywords:** breast cancer, FMO2, SFRP1, prognosis prediction, predictive biomarker

## Abstract

Background: Flavin containing dimethylaniline monoxygenase 2 (FMO2), is downexpressed in diverse tumors and displays vital roles in tumorigenesis. However, the prognostic value and potential mechanism of FMO2 in breast cancer remain unclear.

Methods: The expression of FMO2 was analyzed and the relationship between FMO2 expression level and clinical indicators in breast cancer was analyzed. Then the prognostic value of FMO2 in breast cancer was assessed. The FMO2-correlated genes were obtained, and the highest-ranked gene was chosen. The expression, therapeutic responder analysis, and gene set enrichment analysis of the highest-ranked gene were conducted.

Results: FMO2 was downregulated in breast cancer and was closely related to clinical indicators. Patients with decreased FMO2 expression showed poor overall survival, post-progression survival, relapse-free survival, and distant metastasis-free survival. FMO2 correlates with N/ER/PR subgroups in breast cancer and patients with high FMO2 levels were sensitive to anti-programmed cell death protein 1, anti-programmed death-ligand 1, and anti-cytotoxic T-lymphocyte antigen 4 immunotherapies. Mechanically, FMO2 was positively and highly correlated with secreted Frizzled-related protein 1 (SFRP1), which was downregulated in breast cancer due to hypermethylation. Moreover, SFRP1 was correlated to pathological complete response and relapse-free survival status at 5 years regardless of any chemotherapy, hormone therapy, and anti-HER2 therapy. Gene set enrichment analysis revealed enrichment of component and coagulation cascades, focal adhesion, protein export, and spliceosome.

Conclusions: FMO2 was lower expressed in breast cancer than normal tissues and contributes to subtype classification and prognosis prediction with co-expressed SFRP1.

## INTRODUCTION

Breast cancer has surpassed lung cancer as the most common cancer diagnosed, with an estimated increase of 2.3 million cases [1]. Breast cancer accounts for 25% of cancers in women worldwide [2], and it is also the number one cause of cancer-related deaths among women in the world [3, 4]. Compared with transitioned countries, the mortality rate of breast cancer in transitioning countries is much higher [1]. Although some studies have shown that clinical, pathological indicators and molecular indicators can predict the prognosis of breast cancer [5, 6], early detection, clinical staging, management decision-making and direct treatment of breast cancer are all crucial to the clinical prognosis [7].

Flavin containing dimethylaniline monoxygenase 2 (FMO2), one of the FMO family, is an NADPH-dependent enzyme that catalyzes the N-oxidation of some primary alkylamines through an N-hydroxylamine intermediate [8]. FMO2 prevents cardiac fibrosis via CYP2J3-SMURF2 axis [9], cancer-associated fibroblasts-derived FMO2 acts as a biomarker of macrophage infiltration and prognosis in epithelial ovarian cancer [10]. Although FMO2 is reported to be differentially expressed in tumors, such as early-stage oral squamous cell carcinoma [11], the functional role of FMO2 in breast cancer is largely unknown. Therefore, in the present study, we evaluated the FMO2 expression and its prognostic potential and immunotherapeutic response in breast cancer by comprehensive bioinformatics analysis. In addition, we excavated secreted Frizzled-related protein 1 (SFRP1), a known negative regulator of the Wnt/β-catenin pathway [12], from the perspective of co-expression analysis to reveal the potential molecular mechanism of FMO2 in breast cancer.

## MATERIALS AND METHODS

### FMO2 expression in breast cancer

The FMO2 mRNA expression level was analyzed across all tissues in all available normal and tumor RNA Seq data from Genotype-Tissue Expression (GTEx), The Cancer Genome Atlas (TCGA), and Therapeutically Applicable Research to Generate Effective Treatments (TARGET) through TNMplot [13]. Then the transcript per million of FMO2 was validated in breast invasive carcinoma through UALCAN [14].

### FMO2 expression and different clinical indicators in breast cancer

The expression of FMO2 in different subtypes of breast cancer according to the clinical indicators, including estrogen receptor (ER) status, progesterone receptor (PR) status, human epidermal growth factor receptor 2 (HER2) status, histological types, pathological tumor stage, age status, PAM50 subtypes, triple-negative breast cancer, Ki67 status, Scarff Bloom and Richardson grade status, and Nottingham Prognostic Index status, was analyzed in TCGA (743 breast cancer patients and 92 normal control) and Sweden Cancerome Analysis Network - Breast (SCAN-B) Initiative [15, 16] (3678 breast cancer patients) through Breast Cancer Gene-Expression Miner (bc-GenExMiner) [17].

### Survival analysis

The prognostic value of FMO2 in breast cancer was assessed according to overall survival (OS), post-progression survival (PPS), relapse-free survival (RFS), progression-free survival (PFS), and distant metastasis-free survival (DMFS) using Kaplan–Meier plotter [18], which includes gene chip [19], RNA-sequence [19], protein [20], and immunotherapy [21] from Gene Expression Omnibus, European Genome-Phenome Archive (EGA), and TCGA. Later, the prognostic value of FMO2 in breast cancer was assessed according to OS and DFS among node (N), ER, and PR subgroups in bc-GenExMiner [17] with the TCGA and SCAN-B data. According to FMO2 expression, all patients were divided into high- or low-expression groups, and hazard ratio (HR) with 95% confidence intervals and log-rank P were statistically calculated.

### Correlation analysis

The genes that shared positive and negative correlations with FMO2 in breast cancer were obtained from TNMplot [13] with gene chip data and RNA-sequence data [19]. We then obtained the intersection genes of positive correlation and negative correlation with FMO2 through the Venn diagram, as described previously [22–24], and selected the highest-ranked gene for subsequent analysis. The expression correlation between FMO2 and the highest-ranked gene was verified by bc-GenExMiner [17] with breast cancer DNA microarrays data and RNA-sequence data and UCSC Xena [25] with TCGA breast cancer data. Finally, a density plot was used to show the expression level of two genes in different tissues (normal, tumor, and metastatic tissues).

### SFRP1 expression in breast cancer

The SFRP1 mRNA expression level was analyzed in pan-cancer through TNMplot [13]. And SFRP1 expression in different tissues (normal, tumor, and metastatic tissues) was analyzed. Finally, the SFRP1 mRNA and protein expression levels were validated through UALCAN [14]. The SFRP1 promoter methylation profile was also calculated.

### Therapeutic responder analysis

Subsequently, we used the transcriptomic data of 3104 breast cancer patients to verify the predictive biomarker value of SFRP1 for chemotherapy, hormone therapy, and anti-HER2 therapy through ROCplot [26]. Response to therapy was determined using either author-reported pathological complete response data or relapse-free survival status at 5 years. SFRP1 expression and therapy response are compared using receiver operating characteristics and Mann-Whitney tests.

### Functional analysis

We conducted gene set enrichment analysis (GSEA) with TCGA breast cancer data by LinkedOmics [27] to reduce redundant efforts and focus on the discovery and interpretation of attribute associations.

### Availability of data and materials

All data generated or analyzed during this study are included in this published article and its Supplementary Information Files.

## RESULTS

### FMO2 is downregulated in breast cancer

**Figure 1:**
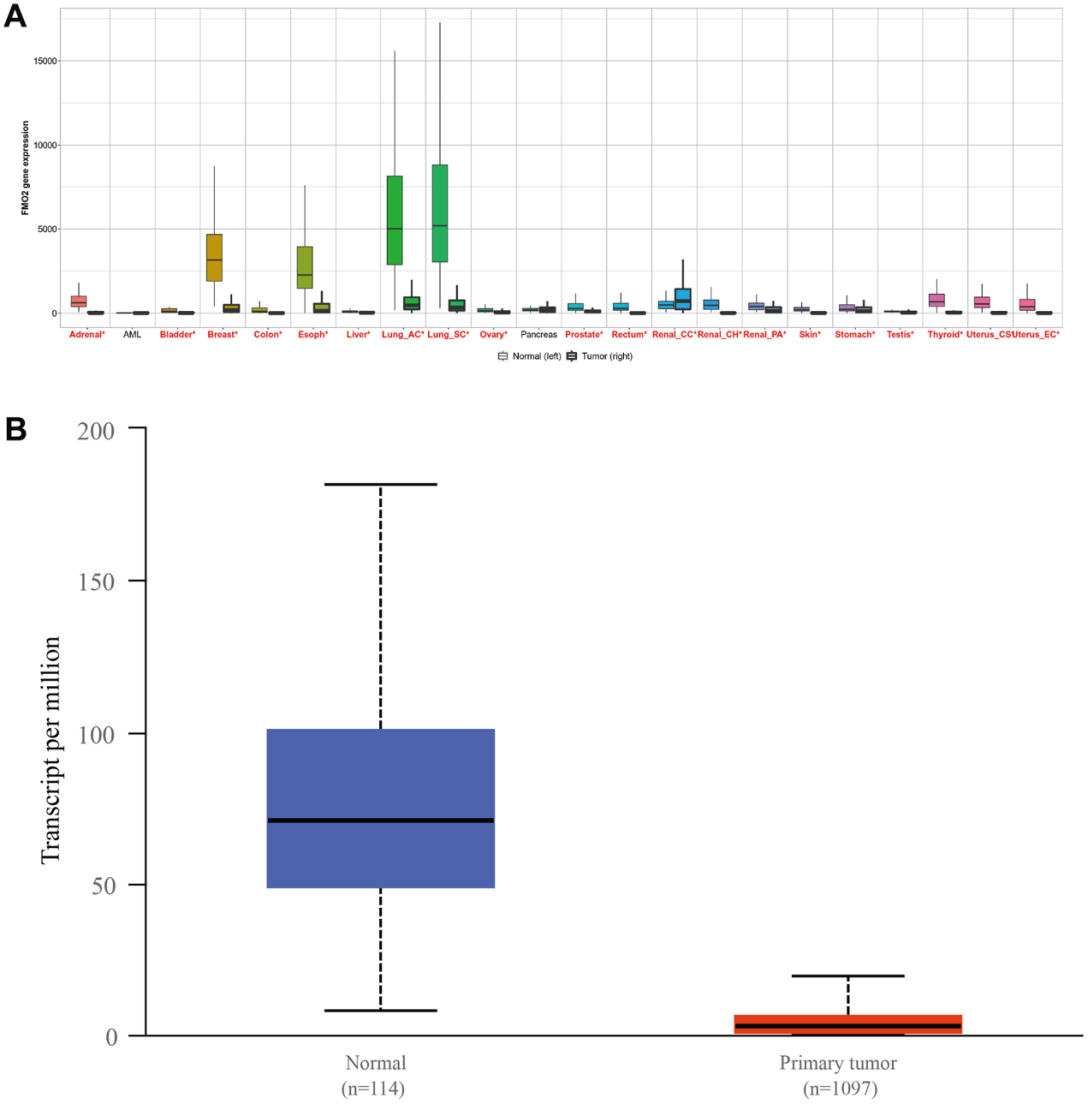
The expression of FMO2 in pan-cancer (**A**) and breast invasive carcinoma, (**B**) P<0.0001.

The pan-cancer analysis indicated FMO2 is dysregulated in various cancers (Figure 1A), including breast cancer. Then TCGA database including 1097 breast invasive carcinoma patients and 114 normal patients showed FMO2 is indeed dysregulated (P < 0.0001) (Figure 1B).

### The relationship between FMO2 expression and clinical indicators in breast cancer

**Figure 2:**
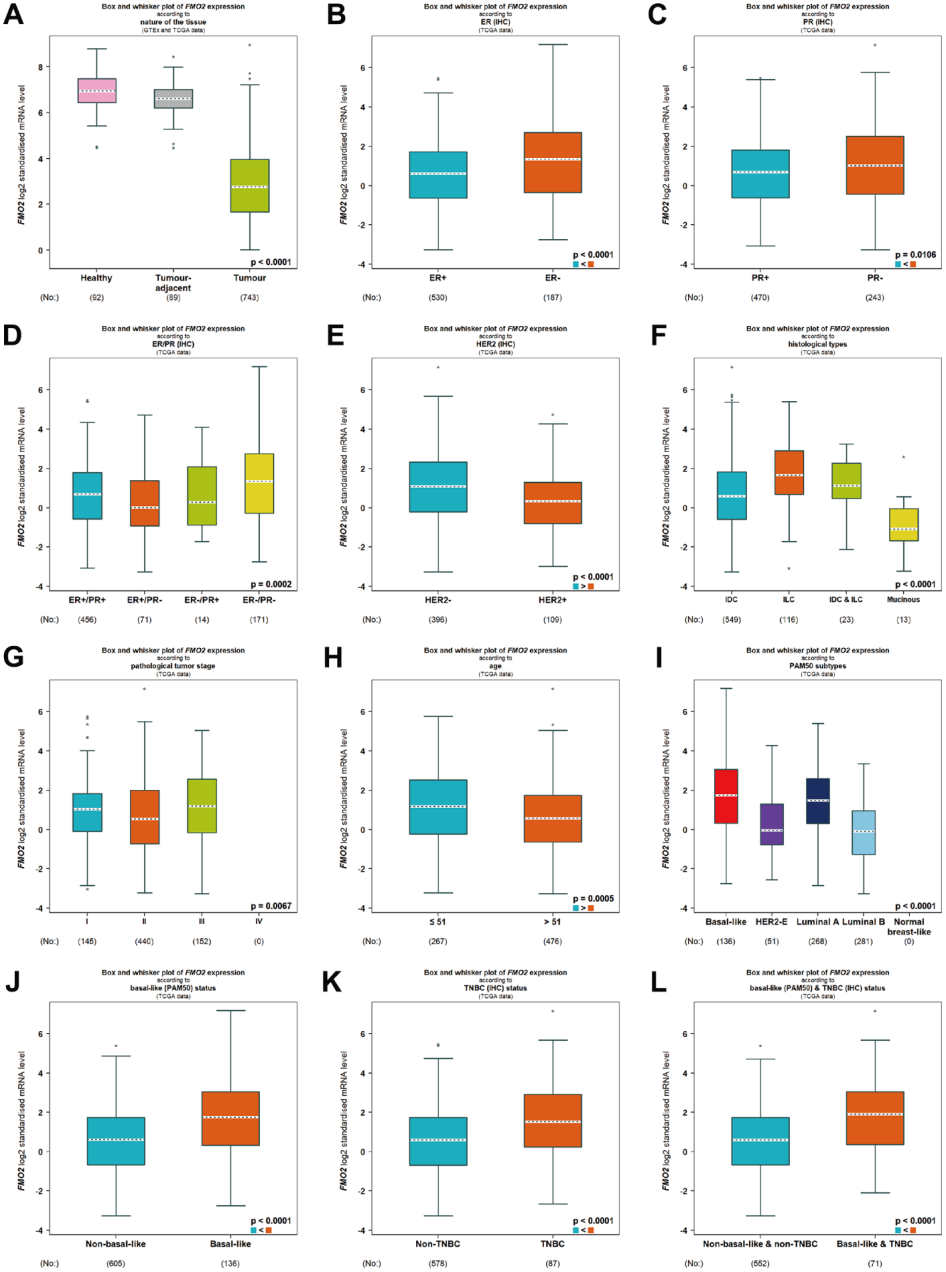
**Relationship between FMO2 and different clinical indicators in TCGA.** (**A**) Nature of the tissue. (**B**) ER status. (**C**) PR status. (**D**) ER and PR status combinations. (**E**) HER2 status. (**F**) Histological types. (**G**) Pathological tumor stage. (**H**) Age status. (**I**) PAM50 subtypes. (**J**) Basal-like (PAM50). (**K**) Triple-negative breast cancer. (**L**) Basal-like (PAM50) and triple-negative breast cancer.

We next compared FMO2 expression in different subtypes of breast cancer according to different clinical indicators. In the TCGA database, FMO2 was downregulated in breast cancer compared to healthy and tumor-adjacent (P < 0.0001) (Figure 2A and Supplementary Table 1). FMO2 was upregulated in ER- and PR- breast cancer compared to those positive (P < 0.0001 and = 0.0106, respectively) (Figure 2B, 2C and Supplementary Table 1). Similarly, FMO2 was upregulated in ER-/PR- breast cancer (P = 0.0002) (Figure 2D and Supplementary Table 1). FMO2 was downregulated in HER2+ breast cancer (P < 0.0001) (Figure 2E and Supplementary Table 1), mucinous histological type (P < 0.0001) (Figure 2F and Supplementary Table 1), and pathological tumor stage II (P = 0.0067) (Figure 2G and Supplementary Table 1). Patients with age less than 51 years old had higher FMO2 expression (P = 0.0005) (Figure 2H and Supplementary Table 1). FMO2 was also dysregulated in PAM50 subtypes (P < 0.0001) (Figure 2I and Supplementary Table 1). FMO2 was upregulated in basal-like (PAM50) (P < 0.0001) (Figure 2J and Supplementary Table 1), triple-negative breast cancer (P < 0.0001) (Figure 2K and Supplementary Table 1), and basal-like and triple-negative breast cancer (P < 0.0001) (Figure 2L and Supplementary Table 1).

In SCAN-B, FMO2 was upregulated in ER-, PR-, and ER-/PR- breast cancer (P < 0.0001, = 0.0006, and < 0.0001, respectively) (Supplementary Figure 1A–1C and Supplementary Table 1), which was consistent with TCGA. In addition, the expression of FMO2 in HER2 status, age status, PAM50 subtypes, and triple-negative breast cancer was also highly consistent with that in TCGA (all P < 0.001) (Supplementary Figure 1D–1I and Supplementary Table 1). FMO2 was dysregulated in Ki67 status, Scarff Bloom and Richardson grade status, and Nottingham Prognostic Index status (all P < 0.0001) (Supplementary Figure 1J–1L and Supplementary Table 1).

### Decreased expression of FMO2 correlates with poor outcomes in breast cancer

**Figure 3:**
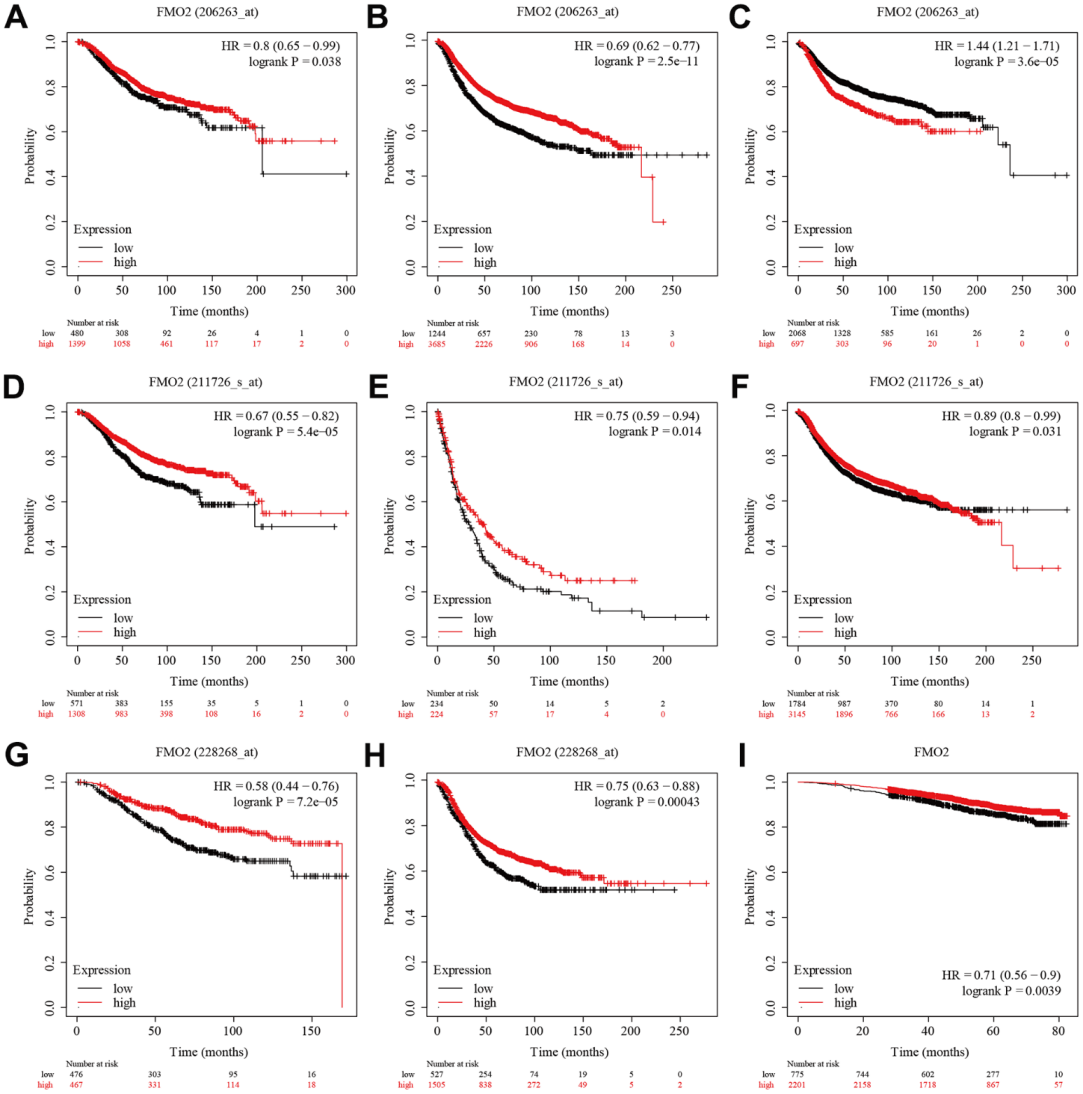
**The survival analysis of FMO2 in the gene chip data and RNA-sequence data of Kaplan–Meier plotter.** (**A**) FMO2 (206263_at) in OS. (**B**) FMO2 (206263_at) in RFS. (**C**) FMO2 (206263_at) in DMFS. (**D**) FMO2 (211726_s_at) in OS. (**E**) FMO2 (211726_s_at) in PPS. (**F**) FMO2 (211726_s_at) in PPS. (**G**) FMO2 (228268_at) in OS. (**H**) FMO2 (228268_at) in RFS. (**I**) FMO2 in OS.

We then analyzed the prognostic value of FMO2. There were three probe data of FMO2 (206263_at, 211726_s_at, and 228268_at) in the gene chip data of Kaplan–Meier plotter. The results indicated that a lower level of FMO2 (206263_at) significantly correlated with poor OS (HR = 0.80 (0.65~0.99), P = 0.038) (Figure 3A) and RFS (HR = 0.69 (0.62~0.77), P < 0.0001) (Figure 3B), while significantly correlated with preferable DMFS (HR = 1.44 (1.21~1.71), P < 0.0001) (Figure 3C). A lower level of FMO2 (206263_at) correlated with poor PPS with no statistical difference (HR = 0.82 (0.62~1.10), P = 0.18) (Supplementary Figure 2A). A lower level of FMO2 (211726_s_at) significantly correlated with poor OS (HR = 0.67 (0.55~0.82), P < 0.0001) (Figure 3D), PPS (HR = 0.75 (0.59~0.94), P = 0.014) (Figure 3E), and RFS (HR = 0.89 (0.80~0.99), P = 0.031) (Figure 3F), while correlated with poor DMFS with no statistical difference (HR = 0.90 (0.77~1.05), P = 0.20) (Supplementary Figure 2B). A lower level of FMO2 (228268_at) significantly correlated with poor OS (HR = 0.58 (0.44~0.76), P < 0.0001) (Figure 3G) and RFS (HR = 0.75 (0.63~0.88), P = 0.00043) (Figure 3H), while correlated with poor PPS (HR = 0.79 (0.55~1.13), P = 0.2) (Supplementary Figure 2C) and DMFS (HR = 0.82 (0.63~1.07), P = 0.14) (Supplementary Figure 2D) with no statistical difference. RNA-sequence in Kaplan–Meier plotter revealed that a lower level of FMO2 significantly correlated with worse OS (HR = 0.71 (0.56~0.90), P = 0.0039) (Figure 3I).

### FMO2 correlates with N/ER/PR subgroups in breast cancer

To further investigate the role of FMO2 in breast cancer OS and DFS, we verified that FMO2 was positively correlated with OS in N+/ER all/PR+ (HR = 0.45 (0.22~0.93), P = 0.0302) (Supplementary Figure 3A), N+/ER+/PR all (HR = 0.48 (0.24~0.96), P = 0.0386) (Supplementary Figure 3B), and N+/ER+/PR+ subgroups (HR = 0.47 (0.22~0.97), P = 0.0411) (Supplementary Figure 3C) and DFS in N+/ER all/PR+ (HR = 0.46 (0.26~0.84), P = 0.0118) (Supplementary Figure 3D), N+/ER+/PR+ (HR = 0.48 (0.26~0.87), P = 0.0159) (Supplementary Figure 3E), N+/ER+/PR all (HR = 0.50 (0.29~0.88), P = 0.0169) (Supplementary Figure 3F), and N+/ER all/PR all subgroups (HR = 0.63 (0.39~1.00), P = 0.0487) (Supplementary Figure 3G) in TCGA from bc-GenExMiner. Similarly, FMO2 was positively correlated with OS in N all/ER+/PR all (HR = 0.65 (0.51~0.84), P = 0.0009) (Supplementary Figure 4A), N all/ER all/PR all (HR = 0.70 (0.57~0.87), P = 0.0015) (Supplementary Figure 4B), N all/ER+/PR+ (HR = 0.67 (0.51~0.88), P = 0.0037) (Supplementary Figure 4C), N-/ER+/PR all (HR = 0.60 (0.43~0.85), P = 0.0042) (Supplementary Figure 4D), N all/ER all/PR+ (HR = 0.70 (0.53~0.92), P = 0.0098) (Supplementary Figure 4E), N-/ER all/PR all (HR = 0.67 (0.50~0.92), P = 0.0115) (Supplementary Figure 4F), N-/ER+/PR+ (HR = 0.64 (0.44~0.92), P = 0.0171) (Supplementary Figure 4G), and N-/ER all/PR+ subgroups (HR = 0.67 (0.46~0.96), P = 0.0287) (Supplementary Figure 4H) and DFS in N all/ER+/PR all (HR = 0.65 (0.51~0.84), P = 0.0009) (Supplementary Figure 4I), N all/ER all/PR all (HR = 0.70 (0.57~0.87), P = 0.0015) (Supplementary Figure 4J), N all/ER+/PR+ (HR = 0.67 (0.51~0.88), P = 0.0037) (Supplementary Figure 4K), N-/ER+/PR all (HR = 0.60 (0.43~0.85), P = 0.0042) (Supplementary Figure 4L), N all/ER all/PR+ (HR = 0.70 (0.53~0.92), P = 0.0098) (Supplementary Figure 4M), N-/ER all/PR all (HR = 0.67 (0.50~0.92), P = 0.0115) (Supplementary Figure 4N), N-/ER+/PR+ (HR = 0.64 (0.44~0.92), P = 0.0171) (Supplementary Figure 4O), and N-/ER all/PR+ subgroups (HR = 0.67 (0.46~0.96), P = 0.0287) (Supplementary Figure 4P) in SCAN-B from bc-GenExMiner.

### Breast cancer with high FMO2 levels was sensitive to immunotherapy

**Figure 4:**
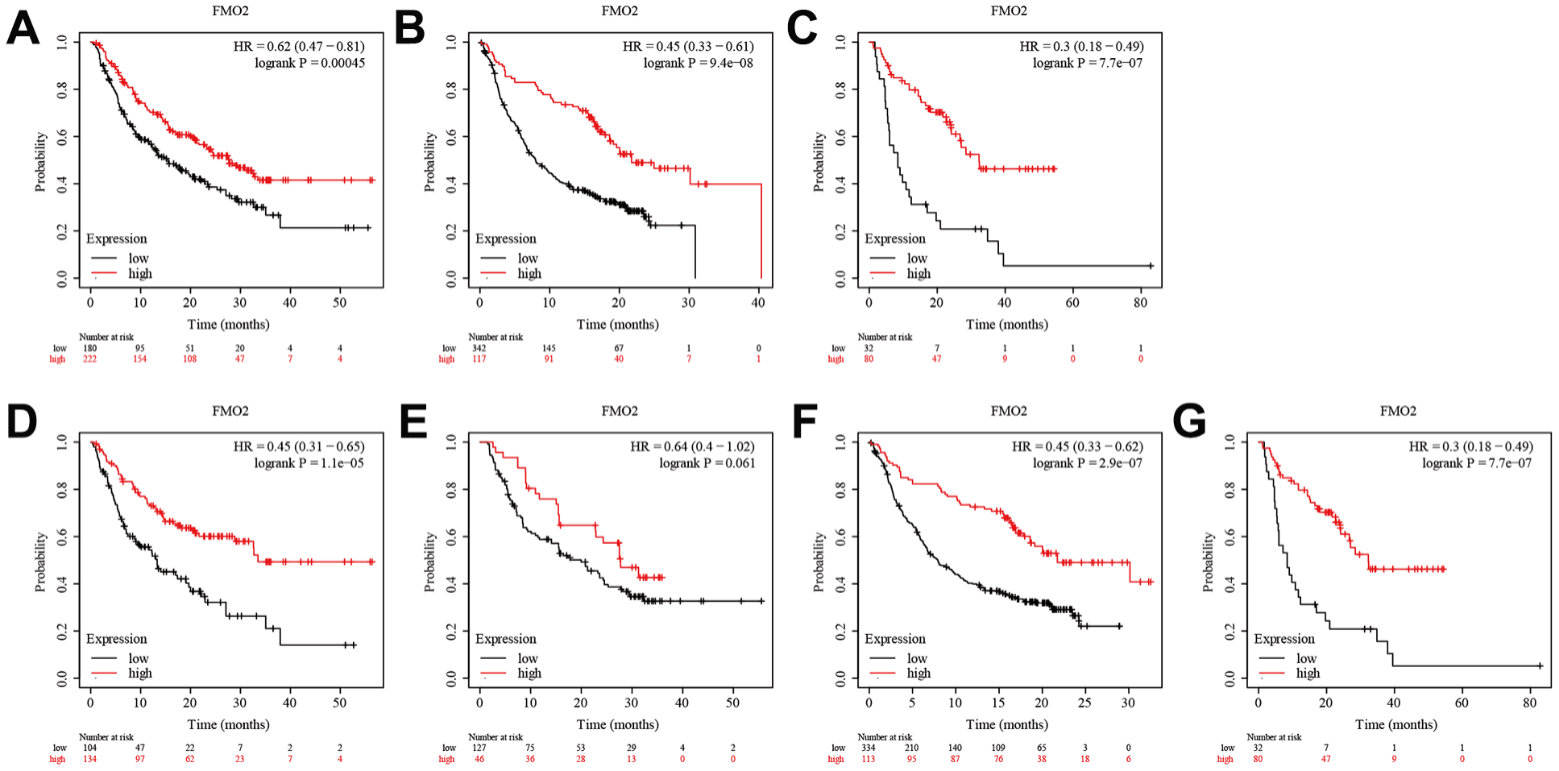
**High FMO2 expression was sensitive to immunotherapy in OS.** (**A**) Anti-PD-1 immunotherapy. (**B**) Anti-PD-L1 immunotherapy. (**C**) Anti-CTLA-4 immunotherapy. (**D**) Pembrolizumab immunotherapy. (**E**) Nivolumab immunotherapy. (**F**) Atezolizumab immunotherapy. (**G**) Ipilimumab immunotherapy.

Anti-programmed cell death protein 1 (PD-1), anti-programmed death-ligand 1 (PD-L1), and anti-cytotoxic T-lymphocyte antigen 4 (CTLA-4) immunotherapies are commonly used in the treatment of various cancers [28, 29]. Here, we analyzed the sensitivity of FMO2 expression to immunotherapy. Analysis of patients receiving anti-PD-1 immunotherapy suggested that patients with high FMO2 expression were more sensitive to anti-PD-1 immunotherapy in OS (HR = 0.62 (0.47~0.81), P = 0.00045) (Figure 4A). Similarly, patients with high FMO2 expression were more sensitive to anti-PD-L1 immunotherapy (HR = 0.45 (0.33~0.61), P < 0.0001) (Figure 4B) and anti-CTLA-4 immunotherapy (HR = 0.30 (0.18~0.49), P < 0.0001) in OS (Figure 4C). Subgroup analyses indicated that patients with high FMO2 expression were more sensitive to pembrolizumab immunotherapy (HR = 0.45 (0.31~0.65), P < 0.0001) (Figure 4D), nivolumab immunotherapy (HR = 0.64 (0.40~1.02), P = 0.061) (Figure 4E), atezolizumab immunotherapy (HR = 0.45 (0.33~0.62), P < 0.0001) (Figure 4F), and ipilimumab immunotherapy (HR = 0.30 (0.18~0.49), P < 0.0001) in OS (Figure 4G). The sample size of patients receiving durvalumab immunotherapy and tremelimumab immunotherapy was too small, so no analyses were conducted.

**Figure 5:**
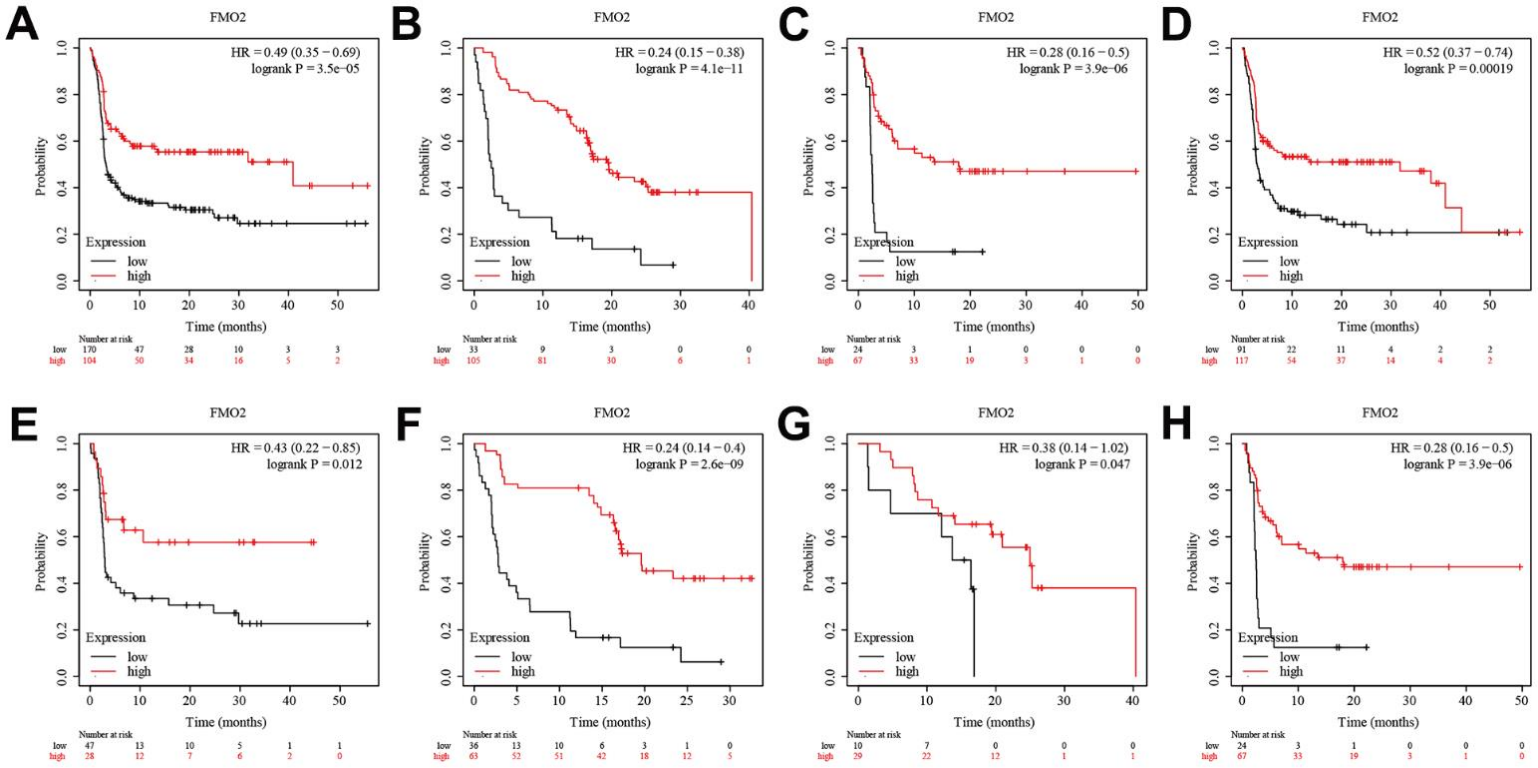
**High FMO2 expression was sensitive to immunotherapy in PFS.** (**A**) Anti-PD-1 immunotherapy. (**B**) Anti-PD-L1 immunotherapy. (**C**) Anti-CTLA-4 immunotherapy. (**D**) Pembrolizumab immunotherapy. (**E**) Nivolumab immunotherapy. (**F**) Atezolizumab immunotherapy. (**G**) Durvalumab immunotherapy. (**H**) Ipilimumab immunotherapy.

Regarding PFS, patients with high FMO2 expression were more sensitive to anti-PD-1 immunotherapy (HR = 0.49 (0.35~0.69), P < 0.0001) (Figure 5A), anti-PD-L1 immunotherapy (HR = 0.24 (0.15~0.38), P < 0.0001) (Figure 5B), and anti-CTLA-4 immunotherapy (HR = 0.28 (0.16~0.50), P < 0.0001) (Figure 5C). Subgroup analyses indicated that patients with high FMO2 expression were more sensitive to pembrolizumab immunotherapy (HR = 0.52 (0.37~0.74), P < 0.0001) (Figure 5D), nivolumab immunotherapy (HR = 0.43 (0.22~0.85), P = 0.012) (Figure 5E), atezolizumab immunotherapy (HR = 0.24 (0.14~0.40), P < 0.0001) (Figure 5F), durvalumab immunotherapy (HR = 0.38 (0.14~1.02), P =0.047) (Figure 5G), and ipilimumab immunotherapy (HR = 0.28 (0.16~0.50), P < 0.0001) in OS (Figure 5H). The sample size of patients receiving tremelimumab immunotherapy was too small, so no analysis was conducted.

### SFRP1 was positively correlated with FMO2 in breast cancer

**Figure 6:**
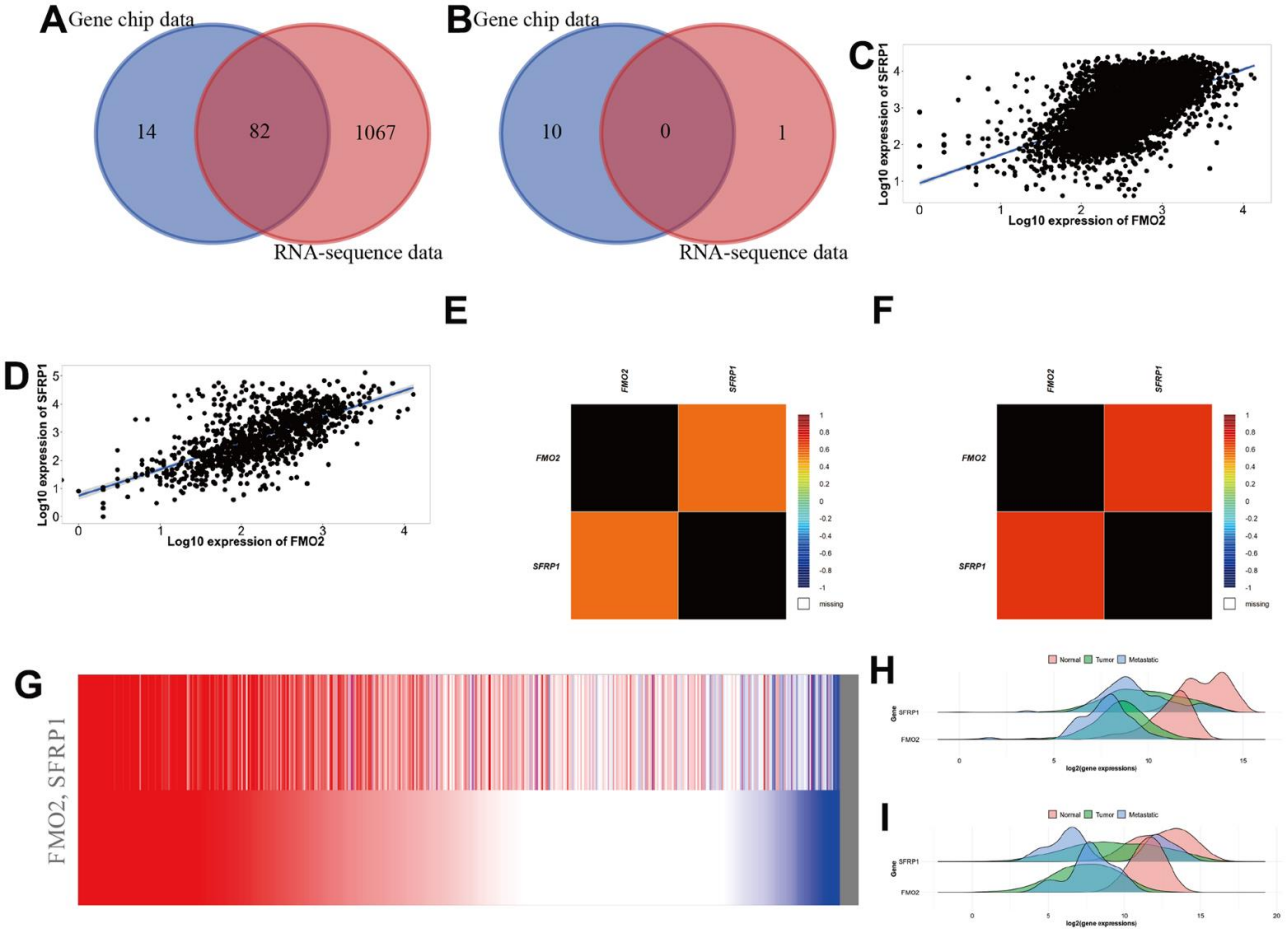
**SFRP1 was positively correlated with FMO2 in breast cancer.** (**A**) Venn diagram of positive correlation genes in TNMplot. (**B**) Venn diagram of negative correlation genes in TNMplot. (**C**) Scatter diagram in gene chip data of TNMplot. (**D**) Scatter diagram in RNA-sequence data of TNMplot. (**E**) Heat map in DNA microarrays data of bc-GenExMiner. (**F**) Heat map in RNA-sequence data of bc-GenExMiner. (**G**) Heat map in TCGA breast cancer data of UCSC Xena. (**H**) Density plot in gene chip data of TNMplot. (**I**) Density plot in RNA-sequence data of TNMplot.

Through TNMplot, we obtained 96 positive correlation genes and 10 negative correlation genes in gene chip data and 1149 positive correlation genes and 1 negative correlation gene in RNA-sequence data, respectively. The top 10 correlation genes are shown in Supplementary Table 2. Then we identified that SFRP1 was positively correlated with FMO2 in breast cancer through the Venn diagram (Figure 6A, 6B) and ranks (Supplementary Table 2). The scatter diagrams showed that SFRP1 was positively correlated with FMO2 in breast cancer in gene chip data (Figure 6C) and RNA-sequence data (Figure 6D). Finally, we validated the co-expression profile of FMO2 and SFRP1 in 8246 patients with breast cancer with DNA microarrays data (Figure 6E) and 4421 patients with breast cancer with RNA-sequence data (Figure 6F) from bc-GenExMiner and 1284 patients with breast cancer with TCGA breast cancer data from UCSC Xena (Figure 6G). The density plots indicated that the expression trend of FMO2 and SFRP1 in the normal, tumor, and metastatic tissues were consistent, regardless of gene chip data (Figure 6H) and RNA-sequence data (Figure 6I).

### SFRP1 was downregulated in breast cancer

The pan-cancer analysis indicated SFRP1 mRNA expression is downregulated in various cancers (Supplementary Figure 5A), including breast cancer. Furthermore, both gene chip data and RNA sequencing data showed that SFRP1 mRNA expression in breast cancer was significantly reduced, and the expression in metastatic breast cancer is lower (Supplementary Figure 5B, 5C). Then TCGA database including 1097 breast invasive carcinoma patients and 114 normal patients showed SFRP1 mRNA expression was indeed downregulated (P < 0.0001) (Supplementary Figure 5D). The Clinical Proteomic Tumor Analysis Consortium (CPTAC) database including 125 breast cancer patients and 18 normal patients showed that SFRP1 protein expression was downregulated (P < 0.0001) (Supplementary Figure 5E).

**Figure 7:**
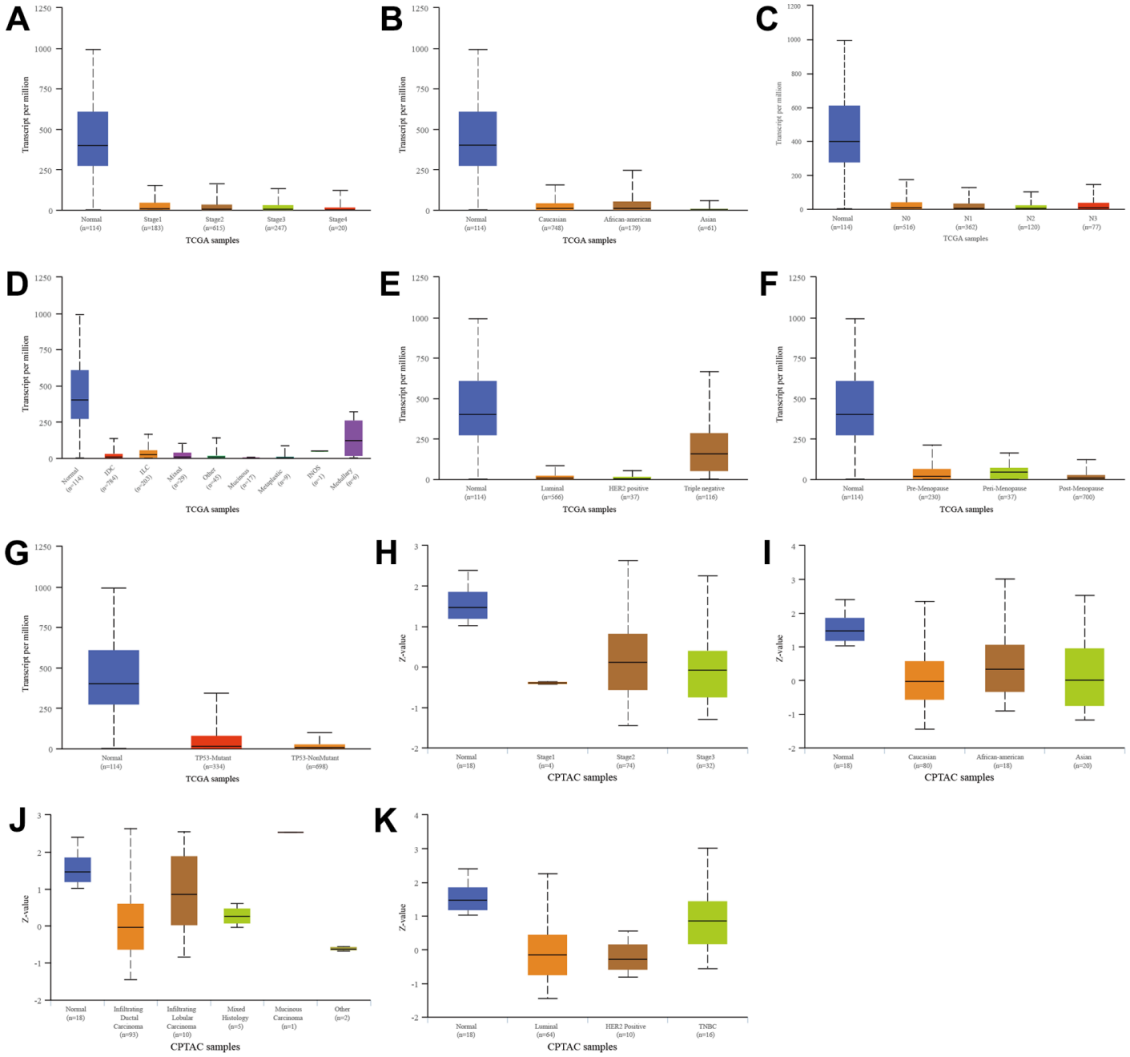
**Relationship between SFRP1 and different clinical indicators.** (**A**) Individual cancer stages in TCGA. (**B**) Race in TCGA. (**C**) Nodal metastasis status in TCGA. (**D**) Tumor histology in TCGA. (**E**) Major subclasses in TCGA. (**F**) Menopause status in TCGA. (**G**) TP53 mutation status in TCGA. (**H**) Individual cancer stages in CPTAC. (**I**) Race in CPTAC. (**J**) Tumor histology in CPTAC. (**K**) Major subclasses in CPTAC.

We next compared SFRP1 expression in different subtypes of breast cancer according to different clinical indicators. In the TCGA database, SFRP1 was downregulated in breast cancer compared to healthy regardless of individual cancer stages (Figure 7A), race (Figure 7B), nodal metastasis status (Figure 7C), tumor histology (Figure 7D), major subclasses (Figure 7E), menopause status (Figure 7F), and TP53 mutation status (Figure 7G). Similarly, In the CPTAC database, SFRP1 was downregulated in breast cancer compared to healthy regardless of individual cancer stages (Figure 7H), race (Figure 7I), tumor histology (Figure 7J), and major subclasses (Figure 7K).

### SFRP1 was hypermethylation in breast cancer

After confirming that SFRP1 was highly expressed in breast cancer, we further explored the SFRP1 promoter methylation level, and the results showed that SFRP1 was hypermethylation in breast cancer (Supplementary Figure 6A), regardless of individual cancer stages (Supplementary Figure 6B), race (Supplementary Figure 6C), nodal metastasis status (Supplementary Figure 6D), tumor histology (Supplementary Figure 6E), major subclasses (Supplementary Figure 6F), menopause status (Supplementary Figure 6G), and TP53 mutation status (Supplementary Figure 6H).

### SFRP1 could act as a predictive biomarker in breast cancer

Through ROCplot, we found that SFRP1 (202037_s_at) was correlated to pathological complete response in breast cancer regardless of any chemotherapy (taxane, anthracycline, ixabepilone, CMF (cyclophosphamide, methotrexate, and fluorouracil), FAC (fluorouracil, Adriamycin, and cytoxan), or FEC (fluorouracil, epirubicin, and cyclophosphamide)) (AUC = 0.578, P < 0.0001) (Supplementary Figure 7A), hormone therapy (tamoxifen or aromatase inhibitor) (AUC = 0.629, P = 0.048) (Supplementary Figure 7B), and anti-HER2 therapy (trastuzumab or lapatinib) (AUC = 0.566, P = 0.047) (Supplementary Figure 7C). Regarding relapse-free survival status at 5 years, SFRP1 could act as a predictive biomarker to predict chemotherapy response (AUC = 0.530, P = 0.130) (Supplementary Figure 7D), hormone therapy response (tamoxifen or aromatase inhibitor) (AUC = 0.604, P < 0.0001) (Supplementary Figure 7E), and anti-HER2 therapy response (trastuzumab or lapatinib) (AUC = 0.516, P = 0.430) (Supplementary Figure 7F).

### SFRP1 regulated breast cancer indecently of the Wnt signal pathway

**Figure 8:**
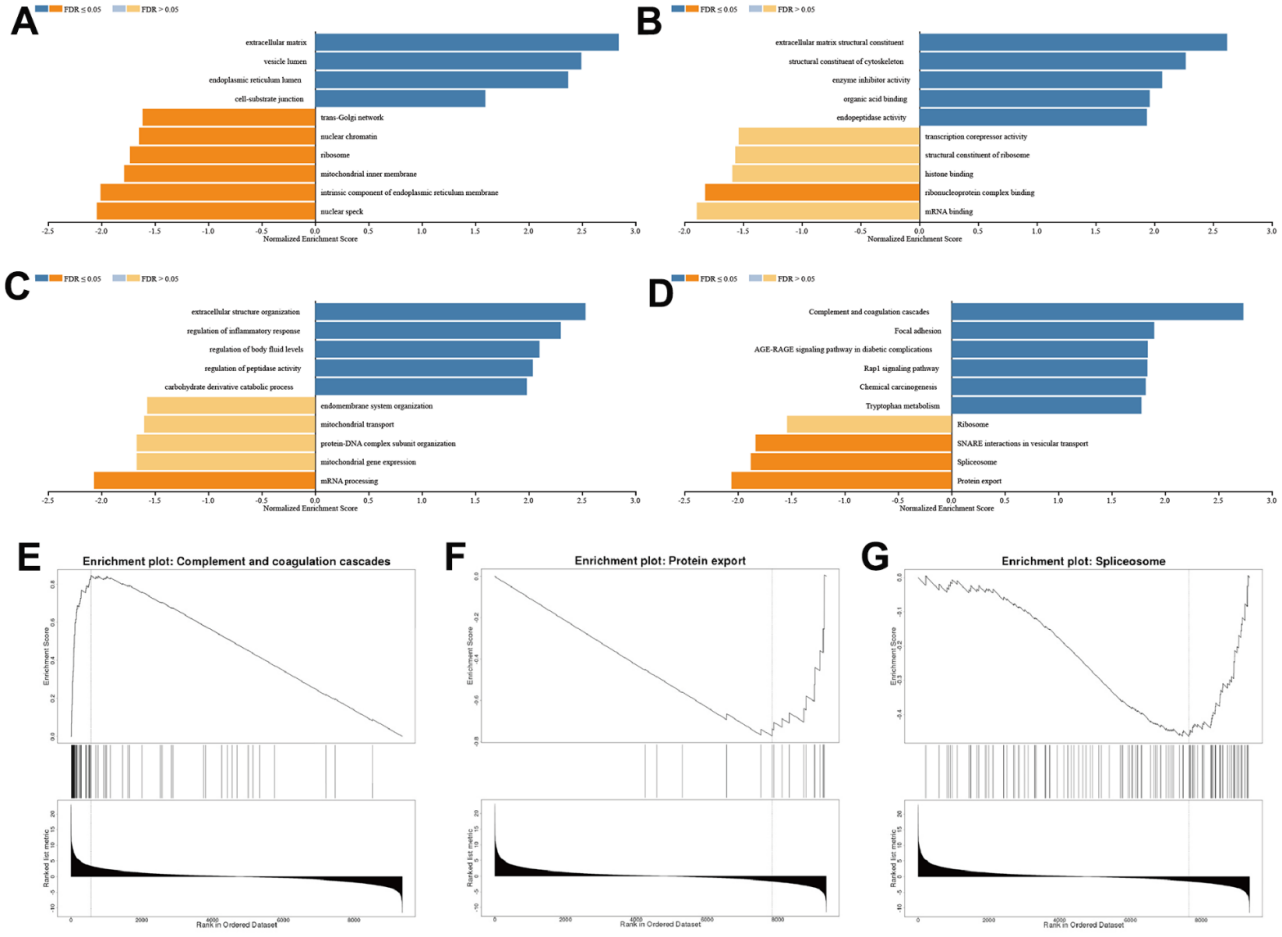
**SFRP1 regulated extracellular matrix in breast cancer.** (**A**) Cellular component. (**B**) Molecular function. (**C**) Biological process. (**D**) Kyoto Encyclopedia of Genes and Genomes pathway. (**E**) Component and coagulation cascades. (**F**) Protein export. (**G**) Spliceosome.

We conducted gene ontology (cellular component, molecular function, and biological process) and Kyoto Encyclopedia of Genes and Genomes pathway analyses. Enrichment results with weighted set cover redundancy reduction indicated that extracellular matrix, vesicle lumen, nuclear speck, and intrinsic component of endoplasmic reticulum membrane were enriched in cellular component (Figure 8A); extracellular matrix structural constituent, structural constituent of cytoskeleton, mRNA binding, and ribonucleoprotein complex binding were enriched in molecular function (Figure 8B); extracellular structure organization, regulation of inflammatory response, mRNA processing, and mitochondrial gene expression were enriched in biological process (Figure 8C); and component and coagulation cascades, focal adhesion, protein export, and spliceosome were enriched in Kyoto Encyclopedia of Genes and Genomes pathway (Figure 8D–8G). In general, SFRP1 predicted the therapeutic response of breast cancer indecently of the Wnt signal pathway.

## DISCUSSION

It is reported that breast cancer is related to abnormal expression of oncogenes [30]. Although the diagnosis, treatment, and prognosis of breast cancer have improved, it is still the most common malignant tumor with the highest incidence rate among women in the world. Identification of new biomarkers of breast cancer is crucial to its diagnosis, treatment, and prognosis. Here, we confirmed that FMO2 is downregulated in breast cancer and FMO2 expression was related to clinical indicators. We later found that decreased expression of FMO2 correlates with poor OS, RFS, PPS, and DMFS. Moreover, FMO2 correlates with N/ER/PR subgroups in OS and DFS. We also explored the reason why upregulated FMO2 expression can benefit breast cancer patients, and the results showed that the high expression of FMO2 may increase the sensitivity of patients to immunotherapy. In addition, correlation analysis showed that SFRP1 is the most potentially related (positively) gene, and more importantly, SFRP1 expression was downregulated in breast cancer patients, which was consistent with FMO2 expression. Then we found that the downregulation of SFRP1 expression may be due to its hypermethylation. SFRP1 was correlated to pathological complete response and relapse-free survival status at 5 years in breast cancer regardless of any chemotherapy, hormone therapy, and anti-HER2 therapy, therefore, SFRP1 could act as a predictive biomarker to predict response among patients with breast cancer. Finally, the functional analysis indicated that SFRP1 might regulate the extracellular matrix to influence component and coagulation cascades, focal adhesion, protein export, and spliceosome pathways in breast cancer.

Up to now, there have been relatively few studies on FMO2 in breast cancer. A new high-throughput drug metabolizing enzymes and transporters microarray platform revealed that single nucleotide polymorphism in FMO2 was significantly associated with docetaxel-induced febrile neutropenia in Lebanese breast cancer patients [31]. FMO2, located within the candidate gains on 1q and is known to be overexpressed in invasive lobular carcinoma relative to invasive ductal carcinoma, which was consistent with our results, with genetic subgroups differed with regard to histology, tumor grading, frequency of alterations, and ER expression [32]. SFRP1 contains a cysteine-rich domain homologous to the putative Wnt-binding site of Frizzled proteins [33, 34]. A previous study found that FMO2 and SFRP1 were under-expressed in ductal carcinoma [35], a common type of breast cancer. Here, we for the first time found SFRP1 positively correlates with FMO2. The promoter hypermethylation is the predominant mechanism of SFRP1 gene silencing in breast cancer [36, 37], importantly, our findings demonstrate this epigenetic mechanism again. Previous studies have shown that SFRP1 can be used as a prognostic marker in breast cancer [36–38] and SFRP1 might be used as a marker for chemotherapy and neoadjuvant chemotherapy response in triple-negative breast cancer [39, 40]. In addition to chemotherapy, we proved that SFRP1 can be used as a predictive marker of hormone therapy and anti-HER2 therapy. SFRP1 interferences with Wnt signaling to block the ligand-receptor interaction [41] or co-regulates with BDNF [42] to have a potential inhibitory effect on breast cancer. Interestingly, we found that SFRP1 affects breast cancer independently of the Wnt signal pathway.

This study has many advantages. Firstly, we proposed for the first time that FMO2 can affect the effect of immunotherapy as a prognostic marker and SFRP1 could be a predictive marker of chemotherapy, hormone therapy, and anti-HER2 therapy on breast cancer. Secondly, we found that FMO2 inhibits breast cancer through co-expression with SFRP1, and the latter plays a role through non-Wnt signal pathway, which is also not reported. Finally, all our research results are basically verified with different data, which makes our research results more robust. Our research also has many areas for improvement. Primally, we confuse all types of breast cancer to solve this huge problem. The prognosis and predictive role of FMO2 and SFRP1 for different types of breast cancer still need further research. In addition, this study is mainly based on existing data and lacks extensive experimental and clinical validation. These markers need more validation before the widespread adoption of treatment decisions for breast cancer.

In conclusion, this analysis revealed that FMO2 was lower expressed in breast cancer compared with normal tissues and contributes to subtype classification and prognosis prediction with co-expressed SFRP1, which could be considered as a predictive biomarker for breast cancer treatment indecently of the Wnt signal pathway by affecting the extracellular matrix.

## Supplementary Material

Supplementary Figures

Supplementary Tables
