# Supplementary material for: Discovery and identification of the prognostic significance and potential mechanism of FMO2 in breast cancer
Source: Aging (Albany NY). 2023 Nov 13;15(21):12651–73. doi: 10.18632/aging.205204 (PMC10683592; doi:10.18632/aging.205204)
Supplement: Supplementary Figures [file aging-15-205204-s001.pdf]

SUPPLEMENTARY FIGURES

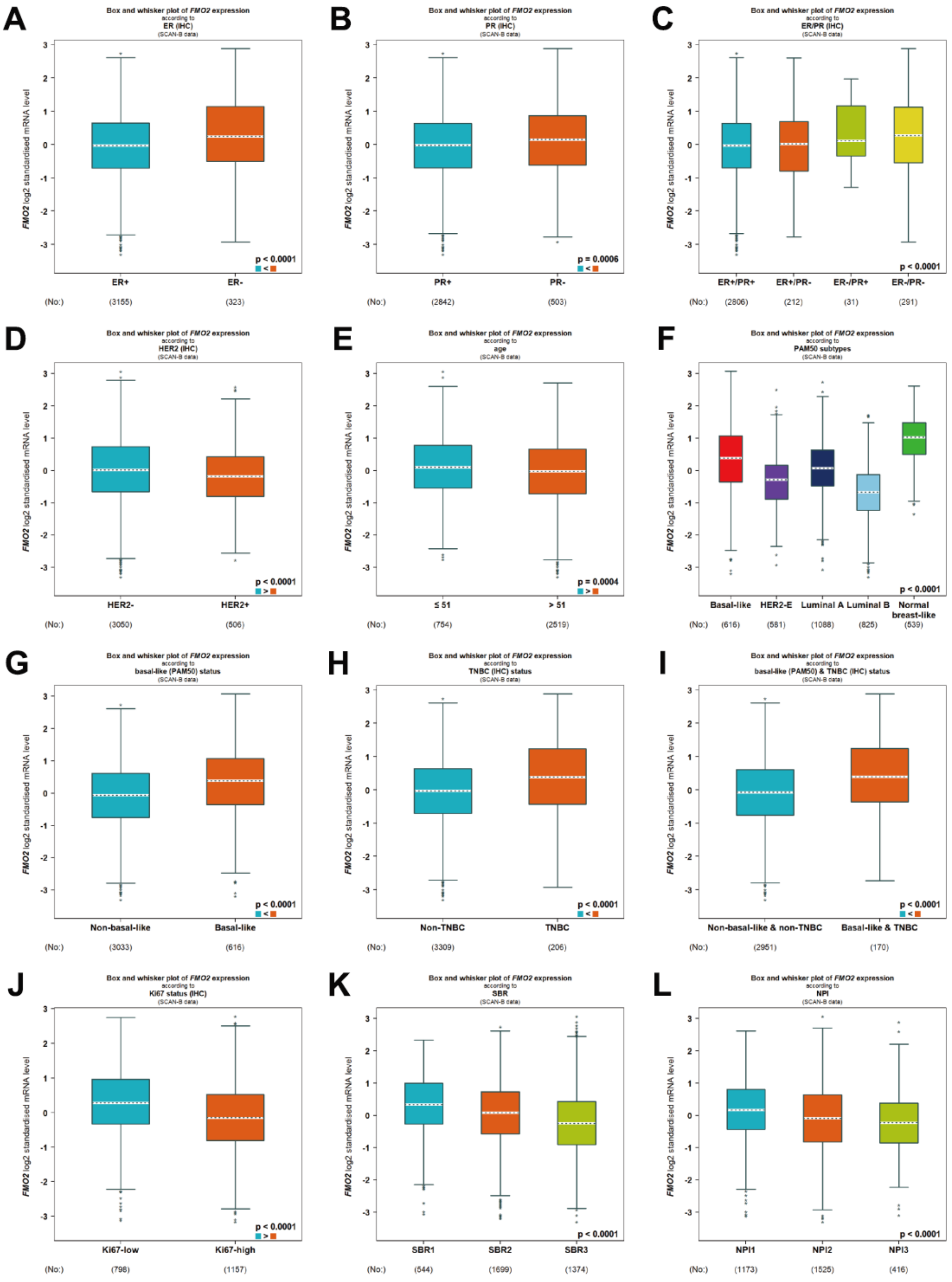

Supplementary Figure 1. Relationship between FMO2 and different clinical indicators in SCAN-B. (**A**) ER status. (**B**) PR status. (**C**) ER and PR status combinations. (**D**) HER2 status. (**E**) Age status. (**F**) PAM50 subtypes. (**G**) Basal-like (PAM50). (**H**) Triple-negative breast cancer. (**I**) Basal-like (PAM50) and triple-negative breast cancer. (**J**) Ki67 status. (**K**) Scarff Bloom and Richardson grade status. (**L**) Nottingham Prognostic Index status.

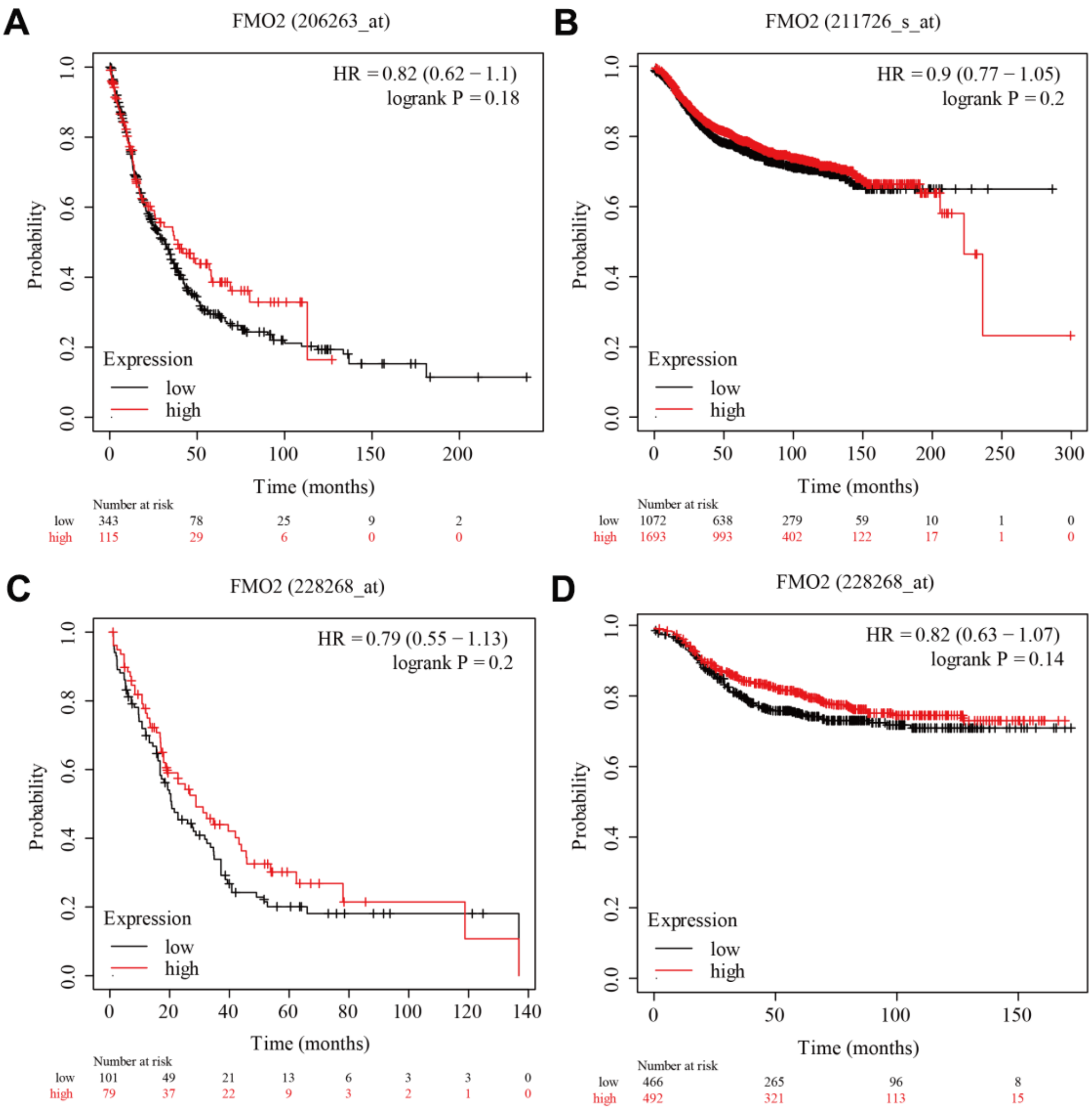

Supplementary Figure 2. The survival analysis of FMO2 in the gene chip data of Kaplan–Meier plotter. (**A**) FMO2 (206263_at) in PPS. (**B**) FMO2 (211726_s_at) in DMFS. (**C**) FMO2 (228268_at) in PPS. (**D**) FMO2 (228268_at) in DMFS.

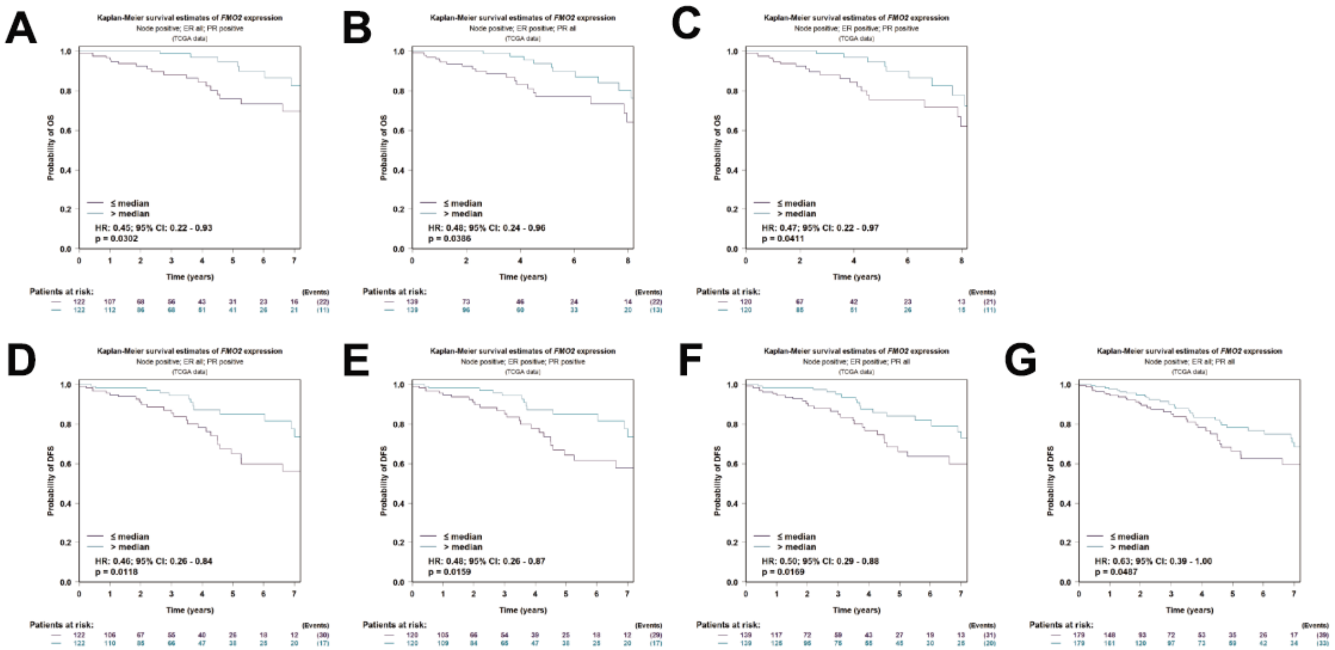

Supplementary Figure 3. The survival analysis of FMO2 in TCGA of bc-GenExMiner. (**A**) OS analysis of N+/ER all/PR+ subgroup. (**B**) OS analysis of N+/ER+/PR all subgroup. (**C**) OS analysis of N+/ER+/PR+ subgroup. (**D**) DFS analysis of N+/ER all/PR+ subgroup. (**E**) DFS analysis of N+/ER+/PR+ analysis. (**F**) DFS analysis of N+/ER+/PR all analysis. (**G**) DFS analysis of N+/ER all/PR analysis.

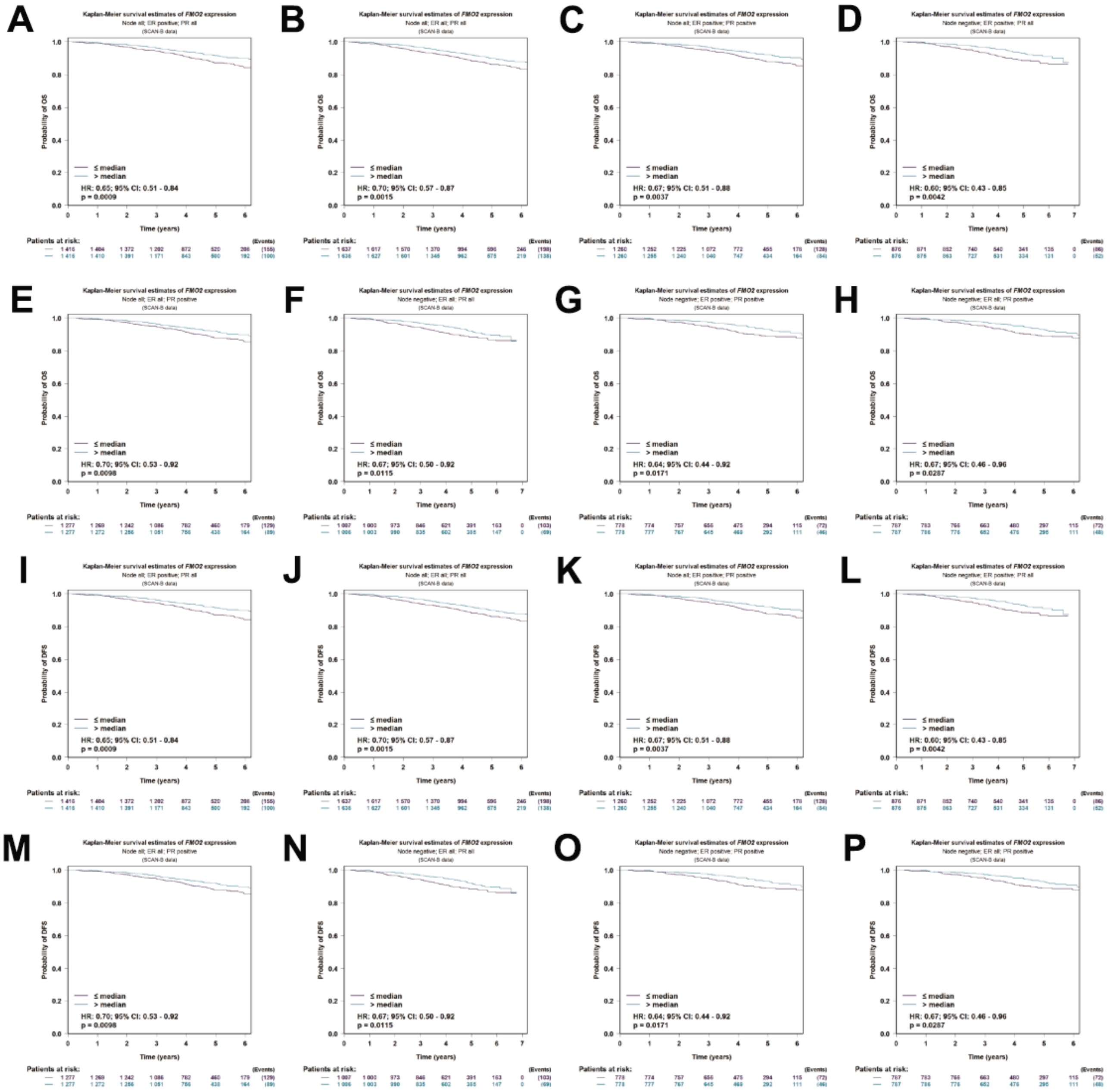

Supplementary Figure 4. The survival analysis of FMO2 in SCAN-B of bc-GenExMiner. (**A**) OS analysis of N all/ER+/PR all subgroup. (**B**) OS analysis of N all/ER all/PR all subgroup. (**C**) OS analysis of N all/ER+/PR+ subgroup. (**D**) OS analysis of N-/ER+/PR all subgroup. (**E**) OS analysis of N all/ER all/PR+ subgroup. (**F**) OS analysis of N-/ER all/PR all subgroup. (**G**) OS analysis of N-/ER+/PR+ subgroup. (**H**) OS analysis of N-/ER all/PR+ subgroup. (**I**) DFS analysis of N all/ER+/PR all subgroup. (**J**) DFS analysis of N all/ER all/PR all subgroup. (**K**) DFS analysis of N all/ER+/PR+ subgroup. (**L**) DFS analysis of N-/ER+/PR all subgroup. (**M**) DFS analysis of all/ER all/PR+ subgroup. (**N**) DFS analysis of N-/ER all/PR all subgroup. (**O**) DFS analysis of N-/ER+/PR+ subgroup. (**P**) DFS analysis of N-/ER all/PR+ subgroup.

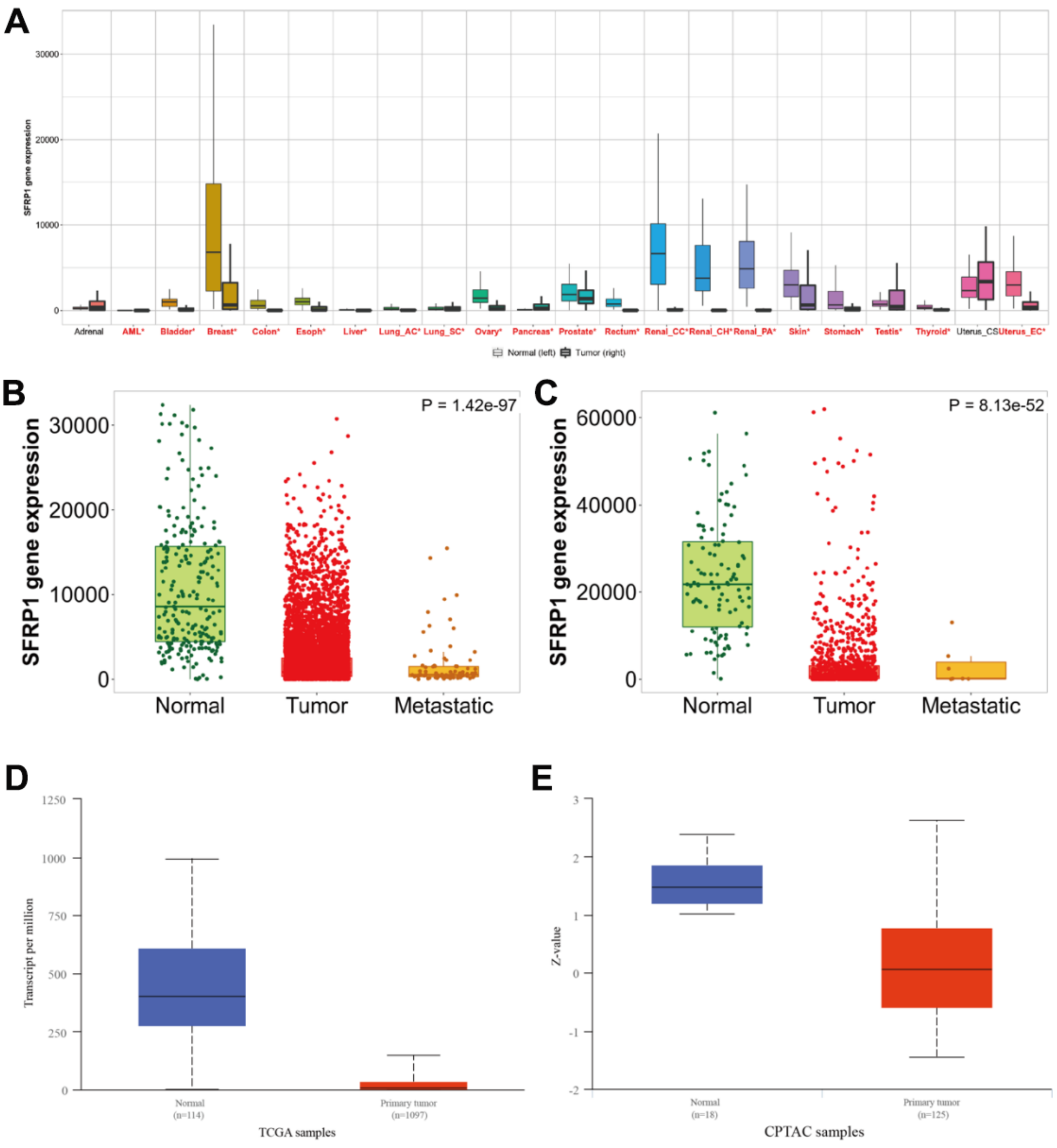

Supplementary Figure 5. The expression of SFRP1. (**A**) SFRP1 mRNA expression in pan-cancer through TNMplot. (**B**) SFRP1 mRNA expression in different breast tissues from gene chip data through TNMplot. (**C**) SFRP1 mRNA expression in different breast tissues from RNA sequences data through TNMplot. (**D**) SFRP1 mRNA expression in breast cancer through UALCAN. (**E**) SFRP1 protein expression in breast cancer through UALCAN.

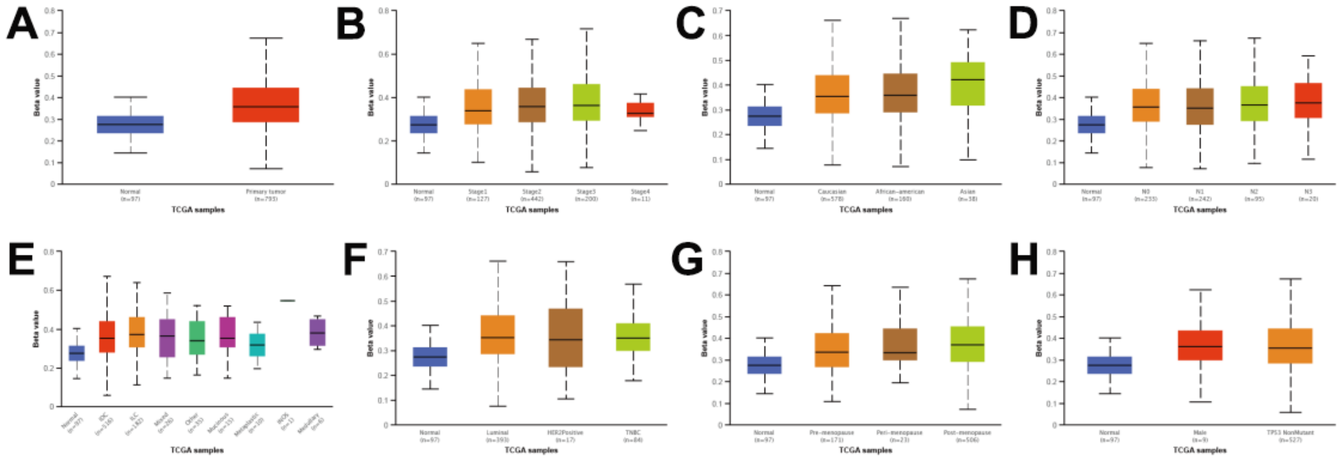

Supplementary Figure 6. SFRP1 was hypermethylation in breast cancer. (**A**) Sample types. (**B**) Individual cancer stages. (**C**) Race. (**D**) Nodal metastasis status. (**E**) Tumor histology. (**F**) Major subclasses. (**G**) Menopause status. (**H**) TP53 mutation status.

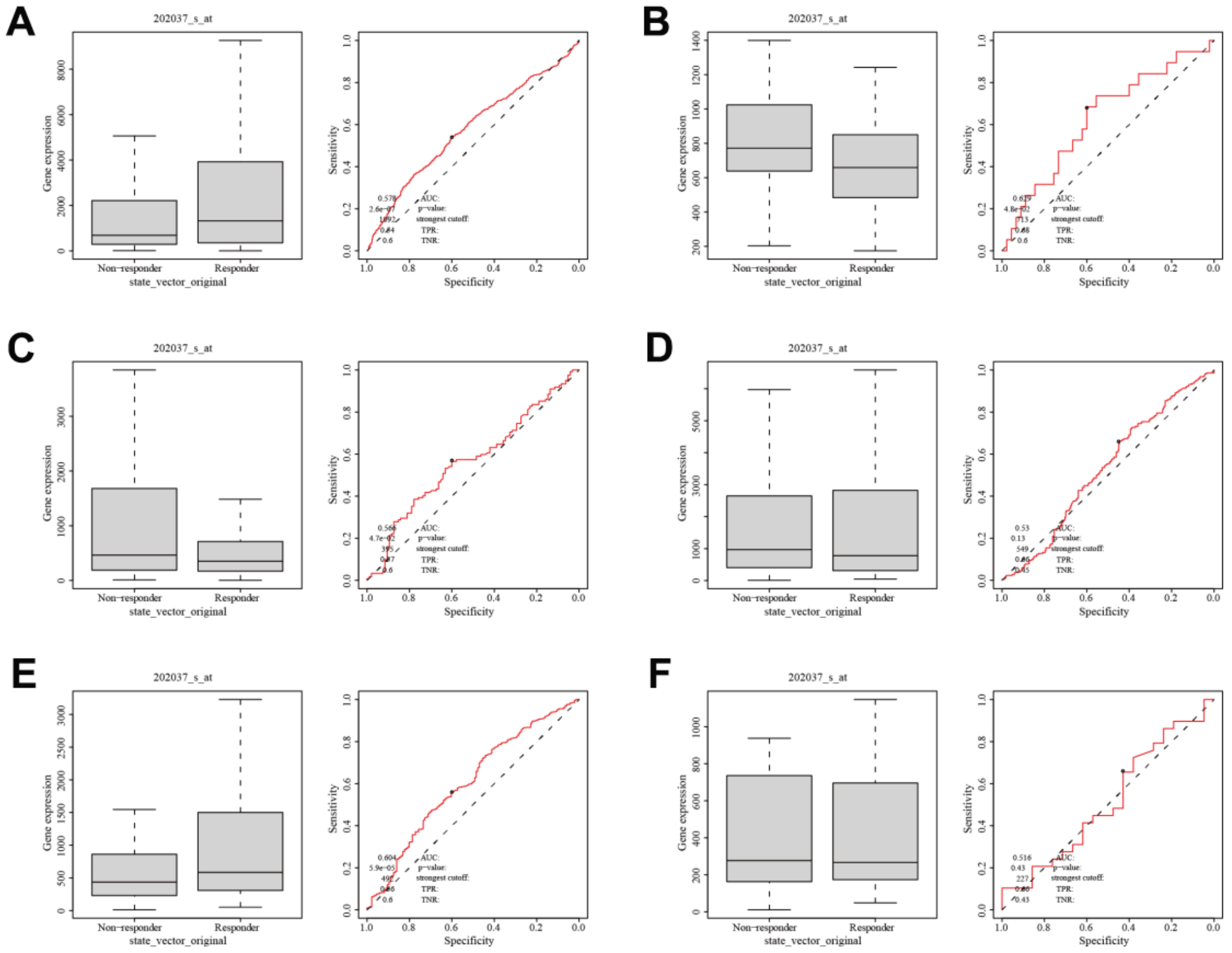

Supplementary Figure 7. SFRP1 could act as a predictive biomarker to predict therapy response. (**A**) Chemotherapy in pathological complete response. (**B**) Hormone therapy in pathological complete response. (**C**) Anti-HER2 therapy in pathological complete response. (**D**) Chemotherapy in relapse-free survival status at 5 years. (**E**) Hormone therapy in relapse-free survival status at 5 years. (**F**) Anti-HER2 therapy in relapse-free survival status at 5 years.
