# Supplementary material for: Discovery and identification of the prognostic significance and potential mechanism of FMO2 in breast cancer
Source: Aging (Albany NY). 2023 Nov 13;15(21):12651–73. doi: 10.18632/aging.205204 (PMC10683592; doi:10.18632/aging.205204)
Supplement: Supplementary Tables [file aging-15-205204-s002.pdf]

SUPPLEMENTARY TABLES

Supplementary Table 1. Relationship between FMO2 and clinical parameters of breast cancer.

Variables	TCGA			SCAN-B		
	Patient number	FMO2 log2 standardized expression	P	Patient number	FMO2 log2 standardized expression	P
**Nature of the tissue**			< 0.0001			
**Healthy**	92	6.8560/ 0.7884				
**Tumor-adjacent**	89	6.5792/ 0.7530				
**Tumor**	743	2.8594/ 1.5755				
**ER status**			< 0.0001			< 0.0001
**ER+**	530	0.6034/1.7245		3155	-0.0411/0.9834	
**ER-**	187	1.3099/1.9443		323	0.2857/1.0738	
**PR status**			0.0106			0.0006
**PR+**	470	0.6576/1.7180		2842	-0.0337/0.9786	
**PR-**	243	1.0391/1.9598		503	0.1435/1.0771	
**ER and PR status combinations**			0.0002			< 0.0001
**ER+/PR+**	456	0.6589/1.7154		2806	-0.0382/0.9787	
**ER+/PR-**	71	0.2465/1.7630		212	-0.0454/1.0285	
**ER-/PR+**	14	0.6162/1.8678		31	0.2993/0.9009	
**ER-/PR-**	171	1.3649/1.9536		291	0.2812/1.0924	
**HER2 status**			< 0.0001			< 0.0001
**HER2-**	396	1.0139/1.7922		3050	0.0203/1.0122	
**HER2+**	109	0.2601/1.6170		506	-0.1702/0.9061	
**Histological types**			< 0.0001			
**IDC**	549	0.6718/1.7653				
**ILC**	116	1.6604/1.5374				
**IDC & ILC**	23	1.0332/1.7440				
**Mucinous**	13	0.8664/1.5635				
**Pathological tumor stage**			0.0067			
**I**	145	0.9529/1.7012				
**II**	440	0.6213/1.8253				
**III**	152	2.5408/1.8054				
**Age status**			0.0005			0.0004
**≤ 51 years old**	267	1.0975/1.8517		754	0.1071/0.9819	
**> 51 years old**	476	0.6083/1.7544		2519	-0.0390/1.0031	
**PAM50 subtypes**			< 0.0001			< 0.0001
**Basal-like**	136	1.6678/1.9598		616	0.3294/1.0238	
**HER2-E**	51	0.2343/1.5514		581	-0.3446/0.7821	
**Luminal A**	268	1.3893/1.5733		1088	0.0291/0.8487	
**Luminal B**	281	-0.1737/1.4615		825	-0.6875/0.8235	
**Normal breast-like**	0			539	0.9795/0.7175	
**Basal-like (PAM50)**			< 0.0001			< 0.0001
**Non-basal-like**	605	0.5807/1.7072		3033	-0.0685/0.9841	
**Basal-like**	136	1.6678/1.9598		616	0.3294/1.0238	
**Triple-negative breast cancer**			< 0.0001			< 0.0001
**Non-TNBC**	578	0.5983/1.7324		3309	-0.0392/0.9805	
**TNBC**	87	1.6091/1.9075		206	0.3626/1.1298	
**TNBC & Basal-like (PAM50)**			< 0.0001			< 0.0001
**Non-basal-like & non-TNBC**	552	0.5724/1.7088		2951	-0.0759/0.9824	
**Basal-like & TNBC**	71	1.7482/1.9278		170	0.3937/1.1218	
**Ki67 status**						< 0.0001
**Ki67-low**				798	0.2500/0.9643	
**Ki67-high**				1157	-0.1350/1.0077	

**Scarff Bloom & Richardson grade status**			< 0.0001
**SBR1**	544	0.3039/0.9245	
**SBR2**	1699	0.0600/0.9757	
**SBR3**	1374	-0.2030/1.0229	
**Nottingham Prognostic Index status**			< 0.0001
**NPI1**	1173	0.1569/0.9412	
**NPI2**	1525	-0.0777/1.0473	
**NPI3**	416	-0.2154/0.9407	

Supplementary Table 2. The top 10 correlation genes with FMO2 in breast cancer.

**Gene chip data**							
**Positive correlations with FMO2**				**Negative correlations with FMO2**			
**Gene symbol**	**Pearson's correlation coefficient**	**P**	**Patient number**	**Gene symbol**	**Pearson's correlation coefficient**	**P**	**Patient number**
SFRP1	0.556	< 0.0001	8245	FAM136BP	-0.5519	< 0.0001	53
SAA2-SAA4	0.542	< 0.0001	481	COX7BP1	-0.4607	2.00E-04	59
KRT16P6	0.5272	< 0.0001	381	SNORD66	-0.4563	6.00E-04	53
LINC02613	0.5268	< 0.0001	381	SNORA2C	-0.4551	6.00E-04	53
KRT17P1	0.512	< 0.0001	381	SNORD99	-0.454	6.00E-04	53
CHL1-AS2	0.5093	< 0.0001	381	DHX40P1	-0.4536	6.00E-04	53
KRT16P2	0.5071	< 0.0001	564	COMMD3-BMI1	-0.4369	< 0.0001	100
CRYAB	0.5059	< 0.0001	8245	FAM138B	-0.4124	< 0.0001	171
SPECC1L-ADORA2A	0.5045	< 0.0001	139	SNORD3A	-0.407	0.0025	53
CHRDL1	0.503	< 0.0001	7950	ELOCP2	-0.4039	< 0.0001	100
**RNA-sequence data**							
**Positive correlations with FMO2**				**Negative correlations with FMO2**			
**Gene symbol**	**Pearson's correlation coefficient**	**P**	**Patient number**	**Gene symbol**	**Pearson's correlation coefficient**	**P**	**Patient number**
CHRDL1	0.6938	< 0.0001	4421	PAFAH1B3	-0.4036	< 0.0001	4421
SFRP1	0.6843	< 0.0001	4421				
IL33	0.6767	< 0.0001	4421				
TSHZ2	0.6742	< 0.0001	4421				
FREM1	0.6739	< 0.0001	4421				
ABCA6	0.6692	< 0.0001	4421				
BOC	0.6672	< 0.0001	4421				
LINC01140	0.6619	< 0.0001	4016				
ABCA8	0.6614	< 0.0001	4421				
ABCA9	0.6588	< 0.0001	4421				
